# Religious celibacy brings inclusive fitness benefits

**DOI:** 10.1098/rspb.2022.0965

**Published:** 2022-06-29

**Authors:** Alberto J. C. Micheletti, Erhao Ge, Liqiong Zhou, Yuan Chen, Hanzhi Zhang, Juan Du, Ruth Mace

**Affiliations:** ^1^ Department of Anthropology, University College London, 14 Taviton Street, London WC1H 0BW, UK; ^2^ Institute for Advanced Study in Toulouse, Université Toulouse 1 Capitole, 1 esplanade de l'Université, 31080 Toulouse Cedex 06, France; ^3^ State Key Laboratory of Grassland and Agro-ecosystems, College of Ecology, Lanzhou University, 222 Tianshui South Road, Lanzhou, Gansu Province 730000, People's Republic of China

**Keywords:** celibacy, institutions, cultural behaviours, parent–offspring conflict, sibling competition, inclusive fitness

## Abstract

The influence of inclusive fitness interests on the evolution of human institutions remains unclear. Religious celibacy constitutes an especially puzzling institution, often deemed maladaptive. Here, we present sociodemographic data from an agropastoralist Buddhist population in western China, where parents sometimes sent a son to the monastery. We find that men with a monk brother father more children, and grandparents with a monk son have more grandchildren, suggesting that the practice is adaptive. We develop a model of celibacy to elucidate the inclusive fitness costs and benefits associated with this behaviour. We show that a minority of sons being celibate can be favoured if this increases their brothers' reproductive success, but only if the decision is under parental, rather than individual, control. These conditions apply to monks in our study site. Inclusive fitness considerations appear to play a key role in shaping parental preferences to adopt this cultural practice.

## 1. Introduction

Institutions are one of the defining features of human societies [1]. Whether institutions are influenced by the inclusive fitness interests of the individuals participating in them is a matter of debate. Some suggest that culture tightly fits ecology [2], whereas others argue that uniquely human cultural transmission mechanisms are necessary and may lead to non-adaptive outcomes [3]. Religious celibacy constitutes an especially puzzling institution, as it both is very costly in terms of fitness and is widespread, being present in virtually all major faith traditions—including Christianity, Buddhism, Hinduism, Jainism and Sufi Islam—in a variety of forms [4]. Why some individuals commit to sexual abstinence is unclear [5]. Some have argued that celibacy is simply the result of a mismatch between our Pleistocene-adapted cognition and life in recently appeared large-scale societies [6]. Others have instead suggested that this and other costly practices led to religious practitioners being perceived as true believers or possessing supernatural powers [7–9]. In this way, they allowed the spread of religious beliefs and associated cooperative norms through cultural group selection [7,10].

Alternatively, lifelong celibacy could be acceptable to families and even encouraged in some sons because it is adaptive, as it increases the reproductive success of the celibate's parents or their families, as speculated by Trivers [11] in his seminal paper on parent–offspring conflict. A predisposition to sacrifice the reproductive potential or even the life of some offspring to favour others may be favoured by selection only in certain conditions and it manifests itself in different ways depending on the ecological and social context [11–13]. Infanticide was common in some hunter–gatherer groups [14], and other means of reproductive suppression are widespread across human societies [15–18] and animal species [19,20], especially in communal breeders like meerkats [21].

Historically, across religious traditions, individuals were often induced by their parents to become monks or nuns at a very young age [4], which suggests that this behaviour could be a form of reproductive suppression. Some have suggested that, by making a fraction of their children celibate, parents in Medieval and Early Modern Europe could avoid dividing their wealth, boosting the survival of the family lineage [22–24]. In addition, owing to their social roles as religious leaders, celibates might have further advanced their families' material interests [22–24]. However, it is unclear whether the practice of inducing a child into celibacy is adaptive, as existing analyses of European genealogical and census data [5,22–24] have not shown whether celibates are associated with higher reproductive success for their parents or siblings. Moreover, while Trivers [11] has argued that parents and their offspring might be in conflict over the decision to become celibate, this behaviour has not been modelled formally and the potential role of demography, including dispersal, remains obscure.

Here, we examine the fitness of families in which some sons become monks, using sociodemographic data from an agropastoralist population in western China, where a significant fraction of males are celibate Buddhist monks. We first investigate the impact of having a celibate brother or son on one's reproductive success. We then develop and analyse a demographically explicit inclusive fitness model [25–31] of lifelong celibacy to identify the selective pressures surrounding this behaviour. We elucidate the inclusive fitness costs and benefits experienced by parents and their sons, assess the scope for parent–offspring conflict and determine under which conditions, if any, celibacy is adaptive. We explore whether these conditions are met in our study population and discuss our empirical results in the light of our mathematical analysis.

## 2. Sociodemographic study

### (a) Study context and history

Our study area is a county in Gansu Province, China, inhabited by Amdo Tibetans whose primary means of subsistence are agriculture, yak and sheep husbandry, and—more recently—wage labour and government benefits. Kinship is patrilineal, and residence is mainly patrilocal [32]. Marriage is now reported as being monogamous, a pattern that might be influenced by current Chinese legislation forbidding polygamy. Rates of polygyny and polyandry are likely to have been higher in the past. Since the late 1980s, a government family planning policy has restricted family size to a maximum of three children (rather than a single child as for Han people in other parts of the country) [33,34]. The level of education is low and primary school attendance became compulsory only in 2000 [35].

Like other parts of China, our study site has witnessed significant social and political changes in the past seven decades, some of which caused significant hardship and starvation, including the Cultural Revolution and ‘The Great Leap Forward’, which have influenced mortality, fertility and reproductive success [36]. In 1958, livestock was redistributed among households to decrease wealth inequality. In 1964, a system of collectives was established, with different families being forced to herd together and resources being divided according in part to a points-based system (determined by how hard an individual was judged to have worked) and in part to original wealth. Collectives were turned into communes in 1968: all livestock were held communally and the production shared according to number of household members and points. In 1981, the Household Responsibility system was introduced: livestock were given to each household to hold privately, while grazing land remained communal. In the early 1990s, land was effectively privatized, divided between households according to the number of members.

Until recently, some families would send a son to the local monastery to become a celibate monk. We have shown elsewhere that, in this population, this is more likely to be a second or later born son; first borns only rarely become monks, as they generally inherit the parental household [37], in line with what has been reported for other Tibetan groups [38]. Stated motives for making a son a monk in Tibetan populations vary. Religious ones include indications from monks or lamas that an ill son should be sent to the monastery if he recovers, the desire to gain religious merit, or the child showing an interest in monks [39]. In other cases, the motives might be economic, such as the inability to support a child because of poverty or the desire to preserve land holdings or livestock herds undivided [39]. There are far fewer nuns than monks in Tibetan Buddhism and they do not enjoy the same level of social recognition as their male counterparts [38]. In our study area, there is currently only one active nunnery (in contrast to seven monasteries) and only five nuns were identified in our sociodemographic survey (in contrast to 268 monks). Monasteries were closed by the government in 1958 and they gradually reopened and experienced a revival in the late 1970s and early 1980s, after the end of the Cultural Revolution in 1976 [40]. Although formal religious activities in this area were suspended for two decades, monasteries play a central role in the life of these populations, who remain on average very religious [40]. There are 16 monasteries in the focal county, seven of which are in the area of the villages we consider. Monks are called by their own or other families to perform religious rituals and are compensated monetarily for these services. Monks study Buddhist texts, pray and carry out a variety of religious ceremonies and rituals for private households or at festivals. In addition to receiving a portion of the donations made by lay people to the monastery, monks are allowed to accept presents of food and money from individuals or households [39].

### (b) Data collection and statistical analyses

Sociodemographic data were collected by L.Z., E.G., Y.C., H.Z. and J.D. in 2017. Ethical clearance was given by the UCL Research Ethics Committee (no. 0449/003) and Lanzhou University. Informed consent was obtained from all participants after they were briefed in the local language. The county where we performed our study comprises 100 villages divided into a number of administrative units. We obtained permission to perform the survey in a third of them, which comprise 21 villages. We surveyed 88.3% of the households in these villages (the remaining 11.7% were not included either because no one was present at home when we visited or, in a small number of cases, because families had relocated to a town).

In total, we collected data from 530 households, which reported on a total of 3591 living and 3378 deceased people, 268 of which were living monks and five living nuns. A household is a group of individuals linked by kinship ties who co-reside or—if monks/nuns—are sons/daughters or brothers/sisters of the head of the household. The head of each household was interviewed and asked about their own, their parents', siblings', spouses’, children and grandchildren's name, age, sex and occupation, in addition to financial information about the household. Married women were asked for a complete birth history. Genealogies were created by linking each person in the census to their mother and father. A person's relatedness to living monks was obtained by matching parental IDs between monks and the focal individual.

We performed four analyses. We constructed multilevel Poisson regressions to explore the possible influence of having a monk brother on a man's or woman's number of children (monk brother analysis and monk sister analysis), and the influence of having a monk son on a man's number of grandchildren (monk father analysis). We further conducted an event history analysis to assess the possible influence of having a monk brother-in-law on a woman's age at first birth (monk sister-in-law analysis). While information about number of children and grandchildren of deceased people could be obtained from their descendants, information regarding their siblings could not always be obtained. For this reason, the monk brother, monk sister and monk sister-in-law analyses are limited to living individuals, whereas the monk father analysis includes both living and deceased individuals. Moreover, each analysis used a different subsample depending on the specific question (data inclusion rules are described in detail in the electronic supplementary material).

In all analyses, we used 10-year birth year cohorts to control for possible differences in fertility and reproductive success due to the historical and political environment experienced by individuals in our sample (see §2a). We also controlled for household wealth, measured as number of yaks. We used current household wealth as our data are not longitudinal, so we do not have information about wealth at the time of the decision to send a son to the monastery. However, private property was reintroduced only in the early 1980s and livestock were distributed equally, according to household size (see §2a), so wealth is less variable over the study period than might be expected in some other contexts. We also controlled for distance from the county capital, and included village as a random effect in all analyses. In addition, we controlled for number of sisters in the monk brother analysis, number of brothers and number of sisters in the monk sister and monk sister-in-law analyses, and number of offspring in the monk father analysis (see the electronic supplementary material for full details). In all cases, we used a model selection approach on sets of *a priori*-defined candidate models, and we chose the best fitting model according to the lowest Akaike's information criterion (AIC) value. Analyses were performed in R (v. 3.5.3) [41] (see electronic supplementary material, information for package information).

### (c) Results

**Figure 1.**
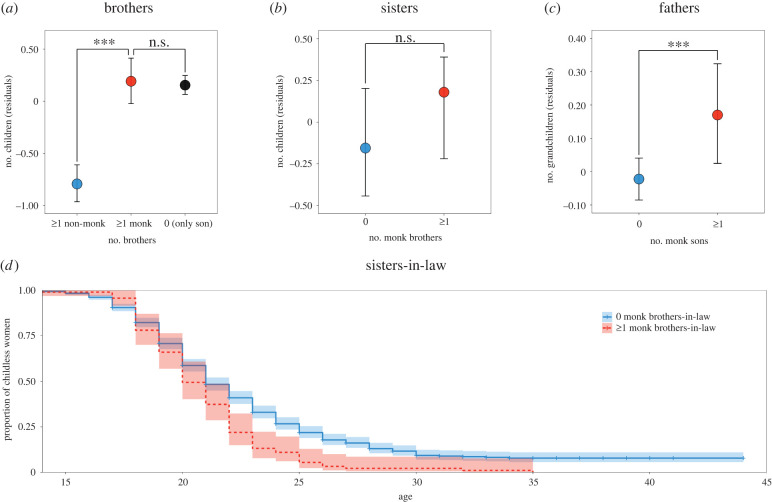
Sociodemographic analysis: reproductive success. Results of the multilevel Poisson regression models indicating that (*a*) men with at least one monk brother have more offspring than men whose brothers are not monks but have similar number of children to only sons; (*b*) women with and without a monk brother have similar numbers of offspring; (*c*) men with a monk son have more grandchildren than those without. Error bars represent 95% confidence intervals calculated by nonparametric bootstrap. (*d*) Kaplan–Meier curve showing that women with at least one monk brother-in-law have an earlier age at first birth than women whose brothers-in-law are not monks. Shaded areas represent 95% confidence intervals. (Online version in colour.)

We first examined the impact of monks on their brothers' reproductive success, starting with men who were about 10 years old when monasteries reopened after the Cultural Revolution in approximately 1980. We found that: men with a monk brother have 1.75 times more children than men whose brothers are not monks (multilevel Poisson regression, *b* = 0.560, 95% CI: [0.330, 0.790], *N* = 934, *p* < 0.001); they have similar numbers of children to only sons (*b* = 0.044, 95% CI: [−0.100, 0.188], *N* = 934, *p* = 0.546; figure 1*a*; electronic supplementary material, tables S1–S4). In this sample, first born sons are more likely to inherit the paternal household and family wealth than second and later born sons [37], in line with Tibetan populations more generally [38]. If we restrict our analysis to first born sons, we find that the effect of having a monk brother remains strongly significant (see electronic supplementary material, tables S5–S8). In neither analysis do household wealth or distance from the county capital have a significant effect. Together, these analyses suggest that monks increase their brothers’ reproductive success by decreasing sibling competition.

We did not find evidence that women with a monk brother have more children than women with only lay brothers (multilevel Poisson regression, *b* = 0.105, 95% CI: [−0.381, 0.591], *N* = 174, *p* = 0.672; figure 1*b*; electronic supplementary material, tables S9 and S10). However, we found greater reproductive success for monk brothers' wives; sisters-in-law of monks have their first child 1.30 years earlier than women who do not have a monk brother-in-law (event history analysis, *b* = 0.265; 95% CI: [0.041,0.490], *N* = 929, *p* = 0.020; average age at first birth = 20.87, s.d. = 3.27; figure 1*d*; electronic supplementary material, tables S11 and S12). This result suggests that the benefit obtained by men from their monk brothers is not accrued through polygamy, but is achieved through earlier reproduction for their wives.

Having shown that men benefit from their brothers being monks, we examined whether it is adaptive for fathers to induce one of their sons to become celibate. We considered men in birth year cohorts before or at 1950, 1951–1960, 1961–1970 and 1971–1980, and focused on their number of grandchildren, as a measure of their reproductive success. Having one fewer potentially reproductive son should result, all else being equal, in a lower number of descendants in the following generation. Instead, we found that men with at least one child who have a monk son have 1.15 times more grandchildren than men with only lay sons (multilevel Poisson regression; *b* = 0.141, 95% CI: [0.042, 0.240], *N* = 2269, *p* = 0.005; figure 1*c* and table 1; electronic supplementary material, table S13). The effect remains significant when restricting the analysis only to men with at least one son (*b* = 0.148, 95% CI: [0.009, 0.287], *N* = 1195, *p* = 0.037; see electronic supplementary material, tables S14 and S15). In both analyses, number of offspring does not have a significant effect on number of grandchildren, and neither do household wealth and distance from the county capital. This result suggests that sending a son to the monastery is not a cost but is instead beneficial to reproductive success for men in this population.

**Table 1.** Reproductive success of monk fathers (best fitting model). Parameter details for the best fitting model of determinants of the number of living grandchildren for 2269 men (Model 3 in electronic supplementary material, table S13). Key variable in bold type.

variable	estimate	95% CI	*Z*-value	*p*-value
(intercept)	1.308	(0.868, 1.747)	5.832	<0.001
ref: ≥1950				
birth year cohort				
1951–1960	0.146	(0.065, 0.227)	3.553	<0.001
1961–1970	0.025	(−0.058, 0.109)	0.591	0.555
1971–1980	−1.498	(−1.693, −1.304)	−15.098	<0.001
wealth	0.027	(−0.004, 0.059)	1.698	0.09
distance to town	−0.012	(−0.021, −0.004)	−2.917	0.004
ref: 1 offspring				
2 offspring	−0.094	(−0.199, 0.010)	−1.775	0.076
3+ offspring	−0.09	(−0.163, −0.017)	−2.415	0.016
ref: 0 monk sons				
**≥1 monk sons**	**0.141**	**(0.042, 0.240)**	**2.79**	**0.005**
random factor Village (variance)	0.001			

## 3. Inclusive fitness model of celibacy

We have shown that religious celibacy appears to be adaptive for the monk's father who encourages and enforces it and for the monk's non-celibate brothers, in an agropastoralist population in western China. We now develop a demographically explicit inclusive fitness model of lifelong celibacy to elucidate under what conditions the behaviour we have described in our sociodemographic study can be favoured by natural selection and to assess potential conflicts of interest within the family. In order to correctly identify the inclusive fitness interests of parents and their sons with respect to the decision to commit to lifelong celibacy, we perform a kin selection analysis using the neighbour-modulated fitness approach [25–31]. We assume that the trait is inherited as if genetically controlled only because this is a tool that allows us to properly assess the inclusive fitness interests of the parties. This is a heuristic model aimed at elucidating the costs and benefits associated with this decision, not a mechanistic model attempting to describe the spread of this trait in a population assuming specific details about its control and expression [25]. We are not claiming that this trait is controlled by a single gene or even that it is influenced by genetics at all. The trait could be—and most likely is—culturally transmitted. The aim of the model is to better understand to what extent it might have been shaped by inclusive fitness interests [42].

We consider a large population subdivided into a large number of groups, each with limited numbers *N*_m_ and *N*_f_ of adult men and women, respectively, and with each woman producing large numbers *K*_m_ and *K*_f_ of sons and daughters, respectively, at the beginning of the life cycle. These simplifying assumptions—shared with numerous population genetic and inclusive fitness models, including Wright's [43] classic infinite island model—abstract away from the complexities of real-world human populations, where families have only a few offspring and may differ in their number and sex. However, they afford mathematical tractability [44] and allow the derivation of analytical results regarding the selective pressures acting on the decision to become celibate and possible conflicts of interest between parents and their sons. We leave the exploration of the potential role of offspring number and configuration on lifelong celibacy to future work adopting different modelling methods.

We assume a life cycle that closely mirrors that of individuals in our sociodemographic study, while retaining mathematical tractability. We consider that, before reaching sexual maturity, boys have the opportunity to commit to lifelong celibacy: the probability that this occurs is our trait of interest, *x*. Then, young adult women and non-celibate young adult men disperse to randomly chosen groups with sex-specific probabilities *d*_f_ and *d*_m_ and, following dispersal, they compete for a limited number of reproductive opportunities. We consider that a focal young adult male participates in competition for reproductive spots with probability *s*(*x*_ind_) = 1 − *x*_ind_, where *x*_ind_ is his probability of becoming a celibate. This means that celibates abstain from participating in this competition (i.e. they always respect their vows of celibacy). Therefore, the marginal cost of celibacy is equal to the partial derivative of the probability of abstaining from competition with respect to one's probability of committing to lifelong celibacy divided by the population average abstinence from competition. In mathematical form, this is given by $c(\bar{x})=-(\partial s(x_{\mathrm{ind}})/\partial x_{\mathrm{ind}})/s(\bar{x})=$ $-(\partial s(x_{\mathrm{bro}})/\partial x_{\mathrm{bro}})\;/\;s(\bar{x})=-(\partial s\;(x_{\mathrm{nbro}})/\;\partial x_{\mathrm{nbro}})/s(\bar{x})=1/(1-\bar{x})$, evaluated at $x_{\mathrm{ind}}=x_{\mathrm{bro}}=x_{\mathrm{nbro}}=\bar{x}$, where *x*_bro_ is the probability of becoming celibate for the focal individual's brothers, *x*_nbro_ for the focal individual's groupmates excluding his brothers, and $\bar{x}$ is the average for the population (notice that, compared with classic models of altruism [25,27,45], we consider additional structuring within the group: brothers versus non-brothers). Non-celibate males then compete for *N*_m_ male adult breeding spots, and females compete for *N*_f_ female adult breeding spots. We formulate a fitness function based on this life cycle and we study how selection acts on celibacy by taking the derivative of fitness with respect to the genic value of a focal individual at a diploid locus controlling celibacy (see electronic supplementary material for fitness function, full model derivation, relatedness coefficients and mathematical demonstrations).

We first consider a case where lifelong celibates simply abstain from competing for reproductive opportunities. We adopt Godfray's ‘battleground’ approach to explore parent–offspring conflict [46,47], that is we identify the optimal strategy first for the offspring and then for his parents. To do this, we hypothetically grant boys—rather than their parents—full control over the decision in order to assess their interests. Analysing the model, including both direct and indirect—i.e. kin-selection—effects [25–31], we obtain that a boy is favoured to increase his probability of becoming celibate when:

3.1 $$-c(\bar{x})+c(\bar{x}){\left(1-d_{m}\right)}^{2}r>0,$$

where $c(\bar{x})$ is the marginal cost of becoming a celibate and *r* is the relatedness between the boy and other boys who were born in his group. That is, by becoming a celibate, the focal male incurs a direct-fitness cost $-c(\bar{x})$ owing to a complete loss of reproductive opportunities (first term in condition (3.1)). He also enjoys a corresponding indirect-fitness benefit owing to decreased competition among other males he is related to. Specifically, the reproductive spot that the focal male might have occupied had he not become celibate and remained philopatric (which occurs with probability 1 − *d*_m_) goes to another male in the group, who is related to the focal male by *r* if he did not disperse (probability 1 − *d*_m_) (second term).

If it is the parents who decide, then they are favoured to increase the probability that a son becomes a monk when:

3.2 $$-c(\bar{x})r_{\mathrm{son}}+c(\bar{x}){\left(1-d_{m}\right)}^{2}r_{\mathrm{songrp}}>0,$$

where *r*_son_ is the relatedness between a parent and their son, and *r*_songrp_ is the relatedness between an adult and boys born in their son's group, including their sons. Notice that this condition has the same structure as condition (3.1), but the relatedness weightings are different.

We find that lifelong celibacy is never favoured by selection, independently of whether the behaviour is under individual or parental control (conditions (3.1) and (3.2) are never satisfied, as 0 ≤ *d*_m_, *r* ≤ 1). This is because the benefit of decreased competition for reproduction is enjoyed by all men in the group—including the celibate's brothers, more distantly related native groupmates, and unrelated migrants—and thus the indirect benefit accrued by or through the celibate does not outweigh the cost of abstinence.

However, if in addition to refraining from reproducing, celibates also increase their brothers' reproductive success, then the situation changes. To explore this possibility, we consider that each non-celibate man's probability of obtaining a breeding spot depends on his competitiveness *t*(*x*_bro_), an increasing function of *x*_bro_, the fraction of his brothers who are celibate (rather than the absolute number, as we are assuming that each woman has a large number of sons). The marginal benefit of celibacy is equal to the partial derivative of competitiveness with respect to one's fraction of celibate brothers divided by the population average competitiveness. In mathematical form, this is given by $b(\bar{x})=(\partial t(x_{\mathrm{bro}})/\partial x_{\mathrm{bro}})/t(\bar{x})=(\partial t(x_{\mathrm{nbro}})/\partial x_{\mathrm{nbro}})/t(\bar{x})$, evaluated at $x_{\mathrm{ind}}=x_{\mathrm{bro}}=x_{\mathrm{nbro}}=\bar{x}$. This benefit can consist in sons obtaining additional family resources (i.e. celibates do not inherit) or enjoying political, social or material support offered by their celibate brothers. Analysing this expanded model, we obtain that a boy is favoured to become a celibate when:

3.3 $$-c(\bar{x})+c(\bar{x}){\left(1-d_{m}\right)}^{2}r+b(\bar{x})(r_{\mathrm{bro}}-{\left(1-d_{m}\right)}^{2}r)>0,$$

where $b(\bar{x})$ is the marginal increase in the competitiveness of the boy's brothers as a result of him becoming a celibate, and *r*_bro_ is the relatedness between the boy and his brothers. That is, by becoming a celibate, the focal male incurs a direct-fitness cost $-c(\bar{x})$ and a corresponding indirect-fitness benefit $c(\bar{x}){\left(1-d_{m}\right)}^{2}r$ (first and second terms in condition (3.3)) as in the abstinence-only case. Moreover, he enjoys an indirect-fitness benefit $b(\bar{x})$ owing to an increase in competitiveness for his lay brothers, who are related to him by *r*_bro_. To this corresponds an indirect-fitness cost $b(\bar{x})$, owing to an increase in competition for all his lay groupmates, who are related to him by *r* if they did not disperse (probability 1 − *d*_m_) and who suffer this disadvantage if the celibate's lay brother benefitting from the competitiveness boost also did not disperse (probability 1 − *d*_m_) (third term in condition (3.3)).

**Figure 2.**
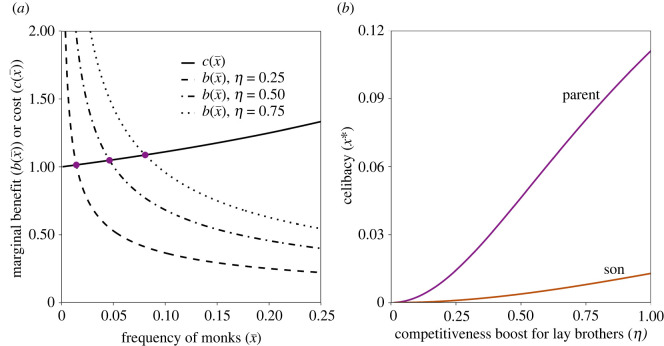
Inclusive fitness model: celibacy. (*a*) Marginal cost of becoming a monk $c(\bar{x})$ (solid line) and marginal benefit $b(\bar{x})$ for three values of *η* (dashed, dot–dashed and dotted lines) as a function of the population frequency of monks, $\bar{x}$. Purple dots represent convergence-stable levels of male celibacy under parental control, i.e. values of x* for which $b(\bar{x})=c(\bar{x})$ evaluated at $\bar{x}=x^{*}$. (*b*) Convergence-stable levels of male celibacy, x*, as a function of the extent of the competitiveness boost for lay brothers, *η*, under parental control (purple) and individual control (brown). For the purposes of illustration, we consider a case where celibates provide benefits to paternal brothers, we consider probability of competing $s(\bar{x})=1-\bar{x}$ and competitiveness $t(\bar{x})=1+\eta^{1/2}$ in both panels, and we assume *d*_m_ = 0.20 and *η* = 0.50 in panel (*a*). (Online version in colour.)

Celibacy can now be favoured by selection. This outcome is the result of frequency dependence: the benefit to lay brothers decreases with the average frequency of celibates in the population, $\bar{x}$. Having a celibate brother results in a considerable competitiveness boost if few men have brothers in the clergy. If the majority of men have celibate brothers, having an additional one has less impact on an individual's chances relative to competitors. In addition, the marginal cost of becoming celibate increases with increasing $\bar{x}$, because the aspiring celibate is renouncing the chance to be one of the increasingly few competitors for reproductive opportunities (figure 2*a*). Furthermore, the probability of becoming celibate favoured by selection increases with the extent of the competitiveness boost to non-celibate brothers. Since celibates pay a high personal cost—forgoing all reproduction—the convergence-stable population frequency of monks remains low even with relatively high competitiveness boosts (figure 2*b*).

The condition for the evolution of monks becomes less stringent if it is parents who decide whether a boy should enter the monastery. Specifically, we find that that the condition for an increase in the probability of becoming a monk is:

3.4 $$\begin{array}{rl} & -c(\bar{x})r_{\mathrm{son}}+c(\bar{x}){\left(1-d_{m}\right)}^{2}r_{\mathrm{songrp}}\\ & \;+b(\bar{x})(r_{\mathrm{son}}-{\left(1-d_{m}\right)}^{2}r_{\mathrm{songrp}})>0, \end{array}$$

which simplifies to $b(\bar{x})>c(\bar{x})$. That is, being equally related to celibate and lay sons, parents commit a son to lifelong celibacy whenever the benefit to the lay son outweighs the cost to the monk (notice that, unlike individual control, in this case the condition does not depend on dispersal or relatedness coefficients). In this way, parents favour their sons to become monks more than the sons themselves would, resulting in parent–offspring conflict (figure 2*b*). The probability of becoming celibate favoured by selection is always higher under parental control than under individual control (see electronic supplementary material for demonstration). While still only a minority of the male population will become celibate, the fraction is potentially much higher under parental than individual control (figure 2*b*).

## 4. Discussion

Taken together, our analyses show that lifelong celibacy can be adaptive under certain conditions. Men with a monk brother have more children and men who sent one of their sons to the monastery have more grandchildren. These effects are strongly significant despite a three-child policy introduced in this area in the late 1980s. With our inclusive fitness model, we have shown that a substantial minority of men can be favoured by selection to be celibate, when the decision is under parental control and when having monk brothers makes men more competitive, leading to higher reproductive success. These conditions are met in our study population, suggesting that this cultural practice has been shaped heavily by the inclusive fitness interests of the monks' parents.

Monks may be enhancing the reproductive success of their brothers in at least two non-mutually exclusive ways. First, as monks do not inherit wealth from their parents [32,38], having a celibate brother might reduce male competition over family resources. We have shown elsewhere [37] that, in this population, men with a monk brother are wealthier than men with a non-celibate brother. Here, we have found that they also have more children, which reveals a key role for brother–brother competition over family wealth. The possibility that monks can also provide material benefits to their brother's family—and thus increase the couple's overall reproductive success—by exploiting their prestigious positions cannot be excluded, as monks command great respect in these communities [38]. We did not find that men with a monk brother have more children than only sons; our analysis of women's age at first birth suggests that their wives might be having children earlier. Further investigation is required to clarify the avenues through which monks benefit their natal families. In other societies, individuals who engage more frequently in religious acts have been shown to have more numerous supportive relations [48,49]. In our case, the fact that only sons and men with a monk brother have the same reproductive success suggests that no such social network effects are present, or that they are unlikely to be important.

Previous research on celibacy suggested that lifelong abstinence could lead to greater lineage survival [5,22–24]. Our sociodemographic analysis has clearly demonstrated that having a celibate child or sibling can be associated with higher reproductive success. Our results help clarify what conditions are necessary for celibacy to appear and be maintained through kin-selected benefits. Genealogical analyses of Medieval and Early Modern European nobility have shown that more children were directed to religious careers in higher social strata [22] and a comparison of two French noble families has suggested that lineages with more celibates were more likely to persist [23]. Both our model and data have shown that celibacy can appear and be maintained in a society without social stratification and hypergamy, two factors that have previously been suggested to be crucial [22,23,50,51]. It has been argued that psychological reinforcement mechanisms and costly ostracism in the case of abandonment of the monastery are key for religious celibacy to appear and be maintained, and they might be used as proximate mechanisms for parents and religious institutions to enforce their own interests [4]. Census data of Catholic priests in ninteenth century Ireland have shown that families who sent at least one son to the seminary were larger, richer and more likely to own land [24]. By contrast, in the present-day United States, Catholic priests tend to come from larger but poorer families [5]. We did not find an effect of wealth in our population: the number of yaks owned by a household does not seem to mediate the effect of having a monk brother or son on reproductive success. Notice, however, that we used current wealth as a proxy for wealth at the time of the celibacy decision, because our data are not longitudinal.

Our model has shown that selection favours celibacy only if it relaxes competition within the monk's family, not within the wider social group. Just like infanticide by parents did not evolve for population regulation [12,52], committing one's son to religious celibacy cannot be favoured by selection for the ‘good of the group’. Moreover, we have shown formally that parents and offspring are indeed in conflict over religious celibacy, with parents favouring higher levels of altruism, analogously to what has been shown for other behaviours subject to parent–offspring conflict [46,47,53–55]. By developing a demographically explicit model employing the latest inclusive fitness methodologies [25,27–31], we have also clarified that dispersal rates influence celibacy decisions when under individual control (and, since only the celibate's brothers benefit from his altruism, celibacy can be favoured even when males never disperse, cf. [25,45,56,57]; see electronic supplementary material for details). On the other hand, dispersal rates have no effect in the more likely scenario when parents decide. In this case, only costs and benefits matter because parents are equally related to all sons, analogously to what has been shown in models of mother–offspring conflict over offspring size [55].

By elucidating the inclusive fitness costs and benefits associated with celibacy, our analysis has highlighted that parent–offspring conflict over the decision is substantial and that only when parents win that conflict would a reasonable proportion of the population become monks. In our population—and in the context of several other religions worldwide [4]—parents induced sons to become monks at a young age, configuring this behaviour as a form of parental manipulation [12]. Several studies have revealed discriminative parental solicitude in a range of contexts that may share similar patterns to the case studied here. Parents often penalize later born sons in regard to care and other investments, including wealth inheritance [14–18,58–61]. For example, Gibson & Gurmu [61] have shown that, in Ethiopia, competition between brothers has adverse effects on later born sons when land is inherited from fathers, but not when it is assigned by the government. In our population, as new and more remunerative job opportunities become available in nearby towns and cities, competition between brothers over family resources may be declining, the incentives for celibacy thus decreasing and parent–offspring conflict gradually disappearing—contributing to a gradual abandonment of the practice.

We have shown that religious celibacy can be adaptive: so why is this practice not more widespread? Two non-mutually exclusive reasons exist. First, as we have discussed above, the conditions for it to be adaptive are not met everywhere or—as could be the case for Europe—were once met but are not any longer. The Tibetan plateau is a harsh environment where competition between siblings for parental resources is likely to be high. Second, religious celibacy as a culturally recognized option needs to be available to a population for it to be adopted as a parental discrimination strategy. In our population, Tibetan Buddhism affords this opportunity. In Medieval and Early Modern Europe, Catholic Christianity also offered this way for parents to suppress their children's reproduction [22,23]. Practices are adopted when they are in line with an individual's interests and, when they are not, they are either abandoned or altered. In this regard, the cases of religions that dropped celibacy requirements for their practitioners—like Protestant Christianity or Japanese Zen Buddhism—are a promising avenue for future research.

Much of the current literature on the evolution of cultural phenomena focuses on transmission biases [62,63], as potential proximate mechanisms for cultural change. However, that framework does not have the power or generality of inclusive fitness theory to help us understand the design and diversity of cultural phenotypes along ecological lines. Humans are strategic in terms of the design or acceptance of cultural traits, adopting those that satisfy preferences that are beneficial to their fitness, as also suggested by recent other work [64–66]. So inclusive fitness is still a framework that potentially has predictive power with respect to the design of cultural phenotypes. Behavioural ecology models have long been used to increase our understanding of the diversity of human behaviour, including cultural behaviour [42], and here we have shown that inclusive fitness interests appear to play a role in shaping both parental preferences and the design of a costly religious institution. Inclusive fitness can help us to make predictions about the phenotypes of cultural institutions that develop in human populations [42].

## Data Availability

Data files for the sociodemographic study are available on the Dryad Digital Repository: https://doi.org/10.5061/dryad.t76hdr83f [67] (we reduced the number of covariates to protect the identity of the participants). Electronic supplementary material, information regarding both the sociodemographic study and the inclusive fitness model, and code used to analyse the data and generate the figures are provided in the electronic supplementary material [68].
