# Correction to: ‘Religious celibacy brings inclusive fitness benefits’ (2022) by Micheletti *et al.*

**DOI:** 10.1098/rspb.2023.1299

**Published:** 2023-06-28

**Authors:** Alberto J. C. Micheletti, Erhao Ge, Liqiong Zhou, Yuan Chen, Juan Du, Ruth Mace

*Proc. R. Soc. B* **289**, 20220965. (Published online 22 June 2022) (https://dx.doi.org/10.1098/rspb.2022.0965)

Here we detail some wording corrections aimed at clarifying our meaning.

(1) In the main text, §2(c), paragraph 1, sentence 4 should read: ‘If we restrict our analysis to male first born children, we find that the effect of having a monk brother remains strongly significant (see electronic supplementary material, tables S5–S8)’. In the electronic supplementary material, the first line of the legends to tables S5–S8 should read: ‘Reproductive success of monk brothers, male first born children’, followed by the relevant reference categories (which remain unchanged). In both cases, ‘male first born children’ replaces ‘first born sons’.
(2) In the main text, §2(c), paragraph 3, sentence 4 should read: ‘Instead, we found that men with at least one child who have a monk son have 1.15 times more grandchildren than men without monk sons’ (where ‘men without monk sons’ replaces ‘men with only lay sons’).
(3) In table 1, the cell in column-1-row-3 should read: ‘ref: ≤ 1950’ (instead of ‘ref: ≥ 1950’)
(4) In the electronic supplementary material, the second sentence of the legend to table S11 should read: ‘The Kaplan–Meier estimated mean age at first birth for women in this sample is 23.1 (s.e. = 0.26). Of the 929 women in this sample, 91 have a monk brother-in-law (estimated mean age at first birth 20.90, s.e. = 0.32) and 838 do not (estimated mean age at first birth 23.4, s.e. = 0.29)’. This sentence replaces confusing summary statistics.

